# The Effects of Lakitelek Thermal Water and Tap Water on Skin Microbiome, a Randomized Control Pilot Study

**DOI:** 10.3390/life13030746

**Published:** 2023-03-09

**Authors:** Bender Tamás, Kalics Gabriella, Árvai Kristóf, Illés Anett, Kósa János Pál, Tobiás Bálint, Lakatos Péter, Papp Márton, Nemes Katalin

**Affiliations:** 1 Polyclinic of the Hospitaller Brothers of St John of God, Árpád Fejedelem Útja 7, H-1023 Budapest, Hungary; 2 Ligetszépe Health Center, Folk College Foundation of Lakitelek, H-6065 Lakitelek, Hungary; gjanics@gmail.com (K.G.); drmedgyesikft@gmail.com (N.K.); 3 Vascular Diagnostics Kft., Lechner Ödön Fasor 3. C. lház. 3. em. 1, H-1095 Budapest, Hungary; kristof.arvai@vascular.hu (Á.K.); janos.kosa@gmail.com (K.J.P.); balint.tobias@vascular.hu (T.B.); lakatos.peter@med.semmelweis-univ.hu (L.P.); 4 Department of Medicine and Oncology, Faculty of Medicine, Semmelweis University, Korányi Sándor u. 2/a, H-1083 Budapest, Hungary; netty.illes@gmail.com; 5 Endocrine Molecular Pathology Research Group, Eötvös Lóránd Research Network, Korányi Sándor u. 2/a, H-1083 Budapest, Hungary; 6 Centre for Bioinformatics, University of Veterinary Medicine Budapest, István u. 2, H-1078 Budapest, Hungary; pappmarci95@gmail.com

**Keywords:** sodium hydrogen carbonate, thermal water, microbiome, next generation sequencing, balneotherapy

## Abstract

The beneficial effects of balneotherapy have been proven by numerous clinical studies on locomotor disorders. To date, there is only scant data on changes in the microbiome system of the skin during balneotherapy. The aim of this study was to compare the effects of thermal water and tap water on the skin’s microbiome in healthy volunteers. 30 healthy female volunteers participated in the study. The experimental group (of 15 women) spent 30-min 10 times, in Gabriella Spring’s thermal baths (i.e., mineral water containing sodium hydrogen carbonate).The controlled group (15 women) had the same, but in tap water. The results of this study have proven that there is a difference in the influencing effects of tap water and medicinal water on the microbiome of the skin. After bathing in the thermal water of Lakitelek, *Deinococcus* increased significantly at the genus level, and the tendency for *Rothia mucilaginosa* bacteria also increased. At the species level, *Rothia mucilaginosa* increased significantly, while *Paracoccus aminovorans* and the tendency for *Paracoccus marcusii* decreased. When the values of the two trial groups after bathing at the genus level were compared, *Rothia bacteria* increased significantly, while *Haemophilus* tended to increase, *Pseudomonas* tended to decrease, *Neisseria* tended to increase significantly, and *Flavobacterium* tended to decrease. At the species level, *Geobacillus vulcani* decreased significantly, and the tendency for *Burkholderia gladioli* decreased. The growth of *Rothia mucilaginosa* and the decrease in the tendency of *Paracoccus, Pseudomonas, Flavobacteroium*, and *Burkholderia gladioli* confirm the beneficial effect of balneotherapy. In this study, trends are represented by the uncorrected *p* value. The main result was that the thermal water changed certain bacteria of the skin, both on the genus and species levels, but there were no significant changes in the tap water used, either at the genus or species level. We first compared the worlds of thermal water and tap water’s microbiome systems. The thermal water decreased the number of certain inflammatory infectious agents and could enhance some of their positive effects, which have been proven at the molecular level. Our results can provide an important clue in the treatment of certain skin diseases. The research of the skin microbiome during balneotherapy can be one of the most intriguing and exciting topics of the future and can bring us closer to understanding the mechanism of action of balneotherapy.

## 1. Introduction

The microbiome is an ecological system comprised of commensal, symbiotic, and pathogenic microorganisms living in the human body. In the framework of the Human Microbiome Project (HMP), systematic research has been ongoing for some time to assess the resident microbial flora normally occurring in various regions of the human outer covering, mainly the skin of the human body, and its associated appendages in various regions [1]. The skin can be considered an ecosystem with an area of 1.8 m^2^ and very diverse areas, which has a significant influence on the microbial flora mapped so far in each area. The flora of the different body regions is well separated, but at the same time relatively uniform within the human population, although there are individual differences between people’s skins. Microbiomes(bacteria, viruses, and fungi) are present everywhere, in and on the body, and although it is often assumed that they are mostly pathogens, they also play many important roles. With increasing urbanization, the proportion of potentially pathogenic taxa of both bacteria and fungi has increased, which may explain the higher prevalence of typical civilization diseases such as acne, atopic dermatitis, and Staphylococcus aureus infections. This clearly shows the influence of the environment and lifestyle on skin diseases [2].

The weight of the microbiome in the human body can be approximately 2.5 kg. The majority of the microbiome can be found in the gastrointestinal tract, while the proportion of those on the skin is smaller. The microbiome can change with age, and the most part of it is harmless; it lives in symbiosis with the host. Their antigenicity is in continuous interaction with the immune system. Based on comparisons to breeding techniques, genomic studies have confirmed the presence of a great deal more bacteria than once believed. Basically, the bacteria found on the skin, similar to the flora of the mucous membrane of digestive organs, can be classified into four large phyla: *Actinomycetes*, *Firmicutes*, *Bacteroidetes*, and *Proteobacteria*—the differences are in the levels of distribution. While *Firmicutes* and *Bacteroidetes* are dominant in the gut, *Actinobacteria* are dominant in the skin. The human skin flora is much more volatile than the intestinal flora throughout a person’s life [3]. The disadvantages of classic culturing are that the tests are significantly influenced by the sampling method and the culture medium used, and some bacteria cannot be grown in vitro. The sequencing-based genomic approach is suitable for determining the exact composition of complex microbial communities without the need to isolate and culture individual components of the community. The tests are based on the fast and High-throughput DNA assays extracted from the communities by using next-generation sequencing (NGS); these extracts were used to examine the genome of the entire community, including non-cultivable microbes.

The scientific field dealing with balneotherapy is called balneology. Balneotherapy means the use of natural mineral waters, natural peloids, which are muds, and natural sources of different gases (CO_2_, H_2_S, radon) for medical purposes: prevention, treatment, and rehabilitation. Balneotherapy can be administered at spas with a special resort environment and atmosphere or elsewhere [4]. Optimally, the effects of balneotherapy are investigated in an outpatient setting without disturbing the patient’s normal lifestyle. Balneotherapy is often used as part of health resort therapy interventions together with massage, exercise, sauna, and other physical therapies, e.g., kinesiotherapy, thermotherapy, electrotherapy, occupational therapy, pharmacotherapy, psychotherapy, nutrition, health education, and cognitive behavioral therapies; relaxation therapies; and complementary therapies. Balneotherapy is a discipline dealing with the effects of medicinal water (*Balneum*, the name of the old Roman baths), which developed mainly in countries rich in thermal water. In addition to the physical properties of water, the absorption of minerals dissolved in the water through the skin can also play a role in its mechanism of action. Balneotherapy has been part of traditional medicine in many European countries for a long time. Health resort medicine includes balneotherapy and other physio therapeutical modalities in a special environment. The chemical effects of balneotherapy are much less clear than the physical ones. There is little evidence for the absorption of mineral elements from water. It can be assumed that the mineral elements deposited in the various layers of the skin during bathing can form deposits and slowly be absorbed through the skin into the circulation, where they exert their effect [5]. Balneotherapy is to be sharply distinguished from today’s fashionable wellness therapies, which involve the short-term bathing of healthy people in tap water or, where applicable, medicinal water without scientific evidence. During wellness therapy (used generally by healthy subjects), people spend a short period in a wellness hotel using thermal facilities (and other paramedical or medical treatments), while balneotherapy (or spa treatment) means a 2 or 3 week long lasting cure. Hydrotherapy is a therapy based on the physical properties of water that is used in almost every country throughout the world, especially for the treatment and rehabilitation of locomotive dysfunctions. Thermal water is not exclusive for that use; tap water may also be used as hydrotherapy. The positive effects of balneotherapy have been proven by numerous clinical studies on locomotor disorders, although there is less evidence for the successful treatment of dermatological and gynecological disorders. Up until now, the main objection against balneotherapy has been the lack of scientific evidence. This fact had indeed remained true for several years, but nowadays it can easily be refuted. If somebody searches in a medical database, such as PubMed, Scopus, or PEDro, he or she can find a growing number of pieces of evidence (including in reviews and meta-analyses) that verify the positive effects of balneotherapy in distinct locomotor problems (especially in OA) and other diseases, so this argument is not as strong as it once was and, in fact, is no weaker than with other well recognized medical interventions [6]. In addition to clinical studies, numerous publications deal with the immunological, antioxidant, and anti-inflammatory effects of medicinal water [7,8]. There is a new breakthrough for investigating possible mechanisms of action in the examination of the skin microbiome in the course of thermal water immersion. 

Hungary has an exceptionally favorable geothermal endowment, as a result of which we are at the forefront of the world in the use of thermal water for medical purposes and the publication of medical studies. In light of this fact, Hungary being a country rich in thermal springs, it is only logical that this type of science is advanced here [9].

The aim of our study was to compare the effects of thermal water with mineral content and tap water on the skin microbiome in healthy volunteers. The study was a randomized controlled trial.

## 2. Materials and Methods

### 2.1. Recruitment of Patients

Healthy volunteers were recruited from the vicinity of the village of Lakitelek, Hungary, and from among those who work there in order to minimize dropouts; there was also a correlating constant medical monitoring during the treatment.

### 2.2. Randomization

Randomization was carried out by an independent person not participating in the study, based on a computerized list.

### 2.3. Inclusion and Exclusion Criteria

Thirty healthy female volunteers participated in the study. The patients had not received balneotherapy treatment for at least 3 months prior to their selection and had transcended the following exclusion criteria: an acute febrile condition, diseases associated with loss of consciousness, psychosis, extensive inflammation of the skin, infectious diseases, coronary disease with resting angina pectoris, unstable angina pectoris, malignant hypertension, severe cardiac decompensation, respiratory disorder, incontinentia alvi et urinae, and acute stages of locomotor diseases (e.g., acute nerve and joint inflammations).

### 2.4. Intervention

The participants (15 women) had 10 times, every consecutive day, 30 min durations in Gabriella Spring’s thermal bath and the control group (15 women) had the same in tap water. The bathing was done at the same time, and the samples were collected before the first treatment and immediately after the last one. The water temperature for both groups was 34 °C. Table 1 shows the anorganic components of the spring.

### 2.5. Method

Samples were collected from the elbow bend of the patient’s body, pre- and post-treatment, with a swab collection and DNA preservation system (i.e., from NorgenBiotek Corp., Thorold, ON, Canada). Microbial DNA was isolated using the Microbiome DNA Isolation Kit (i.e., NorgenBiotek Corp.). The purification process was based on spin column chromatography. Initially, the swab collection tube was incubated with a lysis additive at 65 °C to homogenize samples. The liquid was centrifuged, and the supernatant was transferred to a DNase-free microcentrifuge tube. The lysate, together with the binding buffer, was added and incubated on ice for 10 min. To pellet any cell debris, the lysate was centrifuged for 2 min, after which time the supernatant was collected. Seventy percent ethanol of equal volume was added to the lysate, followed by loading onto a spin-column. The bound portion of DNA was washed using the provided binding buffer and wash solution. The purified DNA was eluted using the elution buffer. The concentration of the isolated DNA was determined by the Qubit dsDNA HS Assay Kit (Life Technologies, Carlsbad, CA, USA). The DNA amplicon library was prepared utilizing the Quick-16S NGS Library Prep Kit (Zymo Research, CA, USA). The kit includes 2 sets of primers that can be used to amplify the corresponding hypervariable regions of the 16S rDNA gene in bacteria: primer sets V1-V2 and V3-V4. The primer sets were paired with Quick-16S™ qPCR premix. After pooling and enzymatic purification of the amplicons, sequencing adapters and sample-specific barcodes were attached to each amplified DNA sample. The concentration of the final library was determined by a qPCR method, and the final purification was carried out with a Select-a-Size MagBead kit (Zymo Research, CA, USA). The sequencing runs were performed using the MiSeq^®^ Reagent Kit v3 (which is a 600-cycle kit made by Illumina, CA, USA).

### 2.6. Bioinformatics and Statistical Methods

Raw reads were quality checked by FastQC [10] and MultiQC [11]. A trimming of the raw reads was performed with Trimmomatic [12] with the following parameters: Leading: 3, Trailing: 20, SlidingWindow: 4:20, Minlen: 50. Dereplication and chimera filtering of the trimmed reads were performed with Vsearch [13]. Reads were classified in taxonomic categories by Kraken2 [14] in the GreenGenes database [15]. Further analysis was performed with the phylo seq [16] and microbiome [17]. R Bioconductor packages in an R [18] environment. Only reads aligning withthe Bacteria kingdom were kept for the analysis. Read counts were processed at the genus and species levels as well. For alpha- and beta-diversity analyses, the read counts were reduced to an even depth. (Depth after rarefication was 32,063 and 3769 at the genus and species levels, respectively.) Alpha-diversity of the samples was assessed by Shannon-diversity andestimated as the mean of 1000 iterations of random rarefications [19]. Bray–Curtis dissimilarity [20] was used for the beta-diversity analysis of the samples and was visualized with NDMS ordination [21]. An analysis of the shifts in bacterial genus and species abundances was performed on the core bacteriome. The core bacteriome was defined as the species/genera present in at least 1% abundance in at least 10% of the samples. DESeq2 was used for the statistical analysis of abundance differences at the core bacteriome level [22]. We have considered notable changes in the tendency of bacterial species/genera when the uncorrected *p*-value was below 0.05.

Three comparisons were made for both the genus- and species level analyses. The first two were paired comparisons analyzing the effects of treatment for patients enrolled in the control and thermal groups, respectively. The third one compared the bacteriome of patients after treatment between the thermal and control groups.

## 3. Results

**Figure 1:**
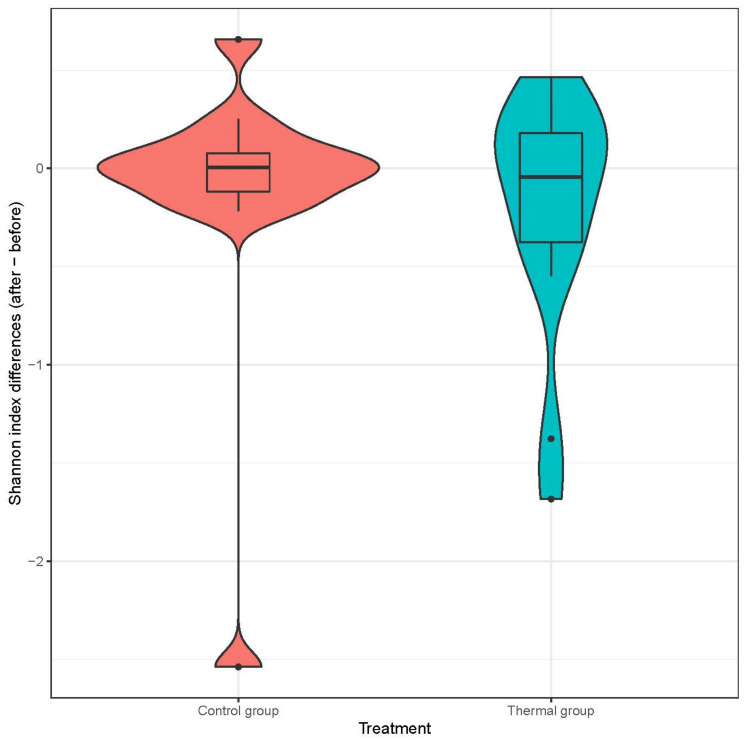
Distribution of differences at Shannon-diversity of patients before and after treatment at the species level. Colors indicate the two treatment groups Control (red) and Thermal water (blue).

**Figure 2:**
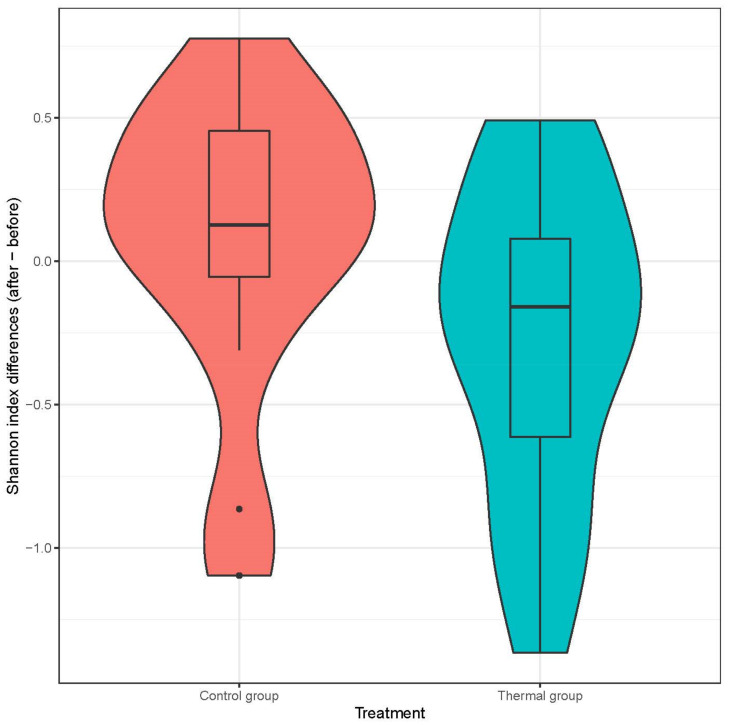
Distribution of differences at Shannon-diversity of patients before and after treatment at the genus level. Colors indicate the two treatment groups Control (red) and Thermal water (blue).

**Figure 3:**
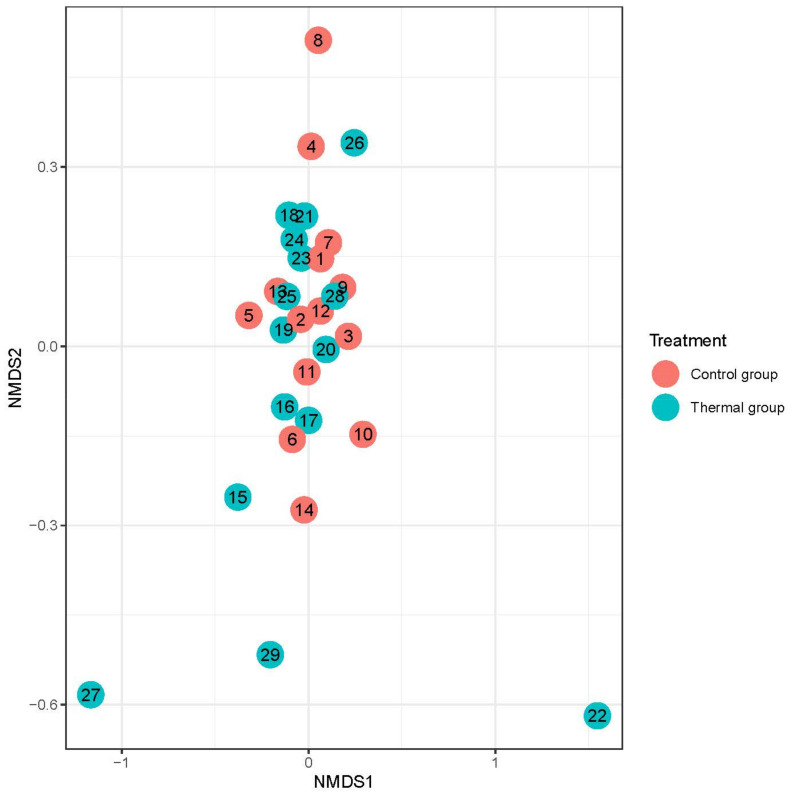
Beta-diversity of patients denoted by Bray-Curtis dissimilarity and presented through NMDS ordination at the species level after treatment. Colors indicate the treatment groups (red = Control, blue = Thermal water).

**Figure 4:**
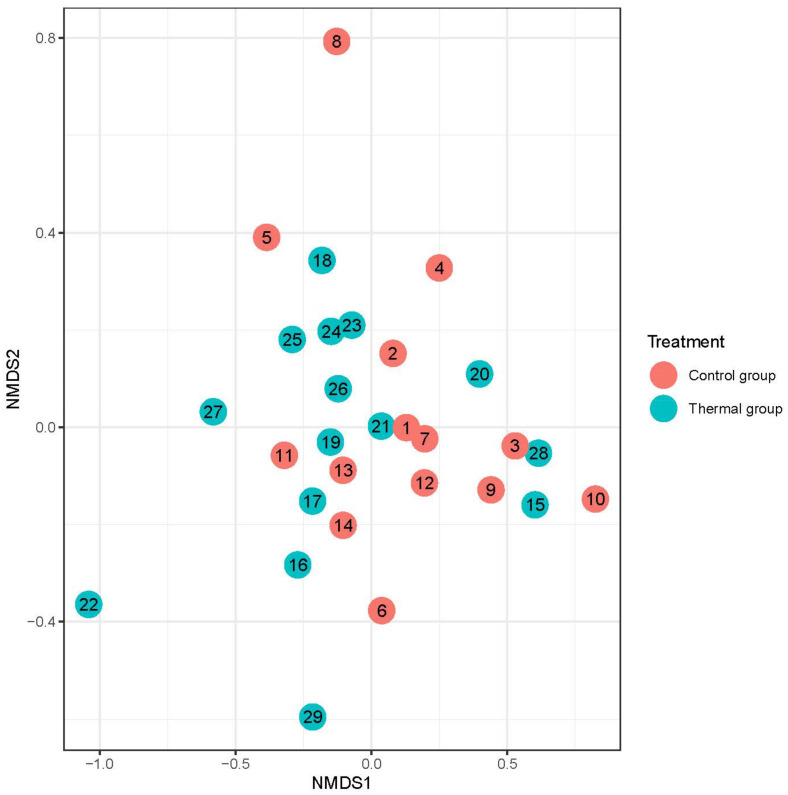
Beta-diversity of patients denoted by Bray-Curtis dissimilarity and presented through NMDS ordination at the genus level after treatment. Colors indicate the treatment groups (red = Control, blue = Thermal water).

**Figure 5:**
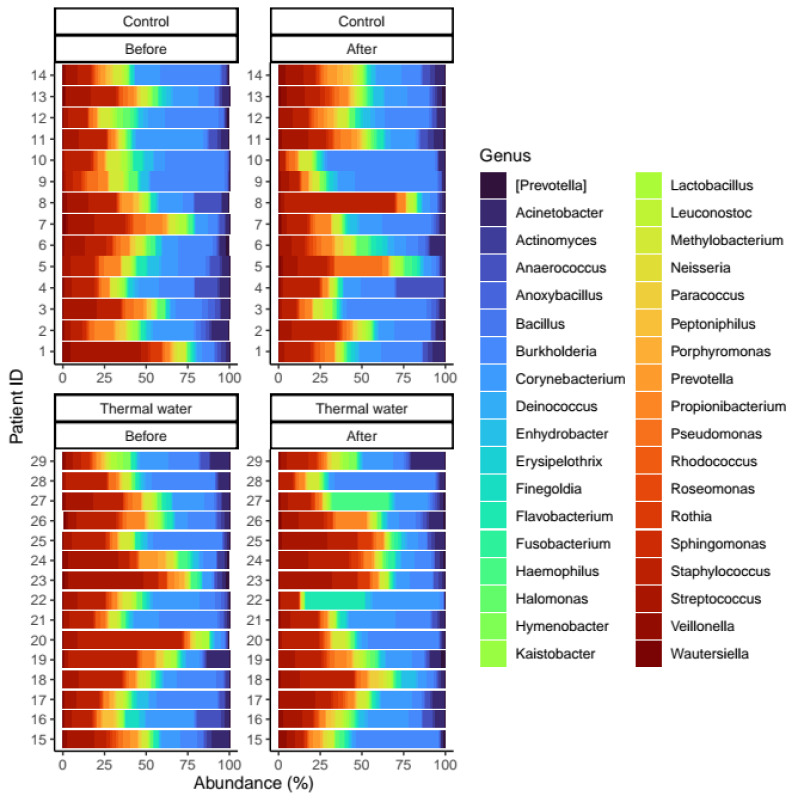
Relative distribution of the core genera found in our analysis. Separate quarters indicate the data from patients before (Before) and after (After) the treatment for each groups (Control and Thermal water groups, respectively).

**Figure 6:**
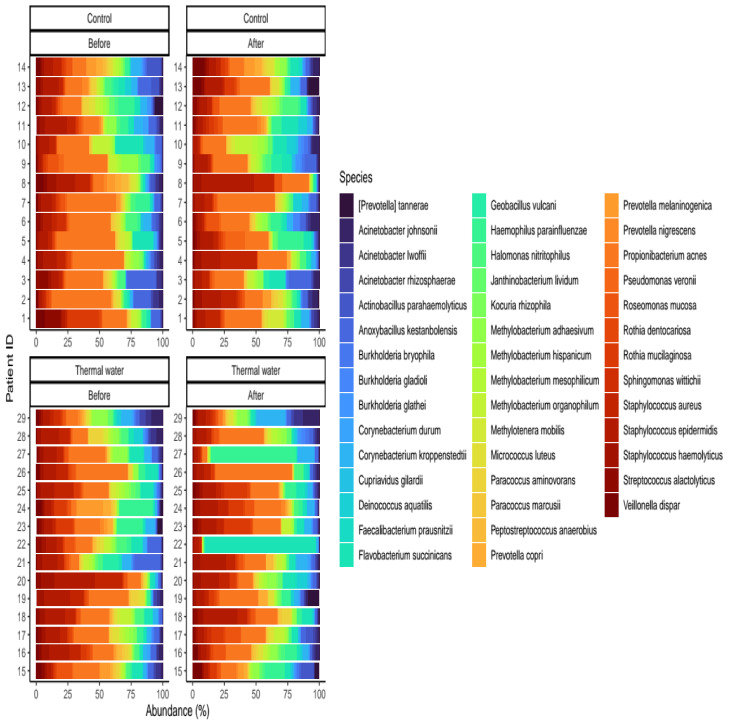
Relative distribution of the core species found in our analysis. Separate quarters indicate the data from patients before (Before) and after (After) the treatment for each groups (Control and Thermal water groups, respectively).

We evaluated 30 healthy volunteers’ data.(With the mean age being 43 and the BMI value being in the normal range).Comparing the thermal water before and after bathing on the genus level, *Deinococcus* increased significantly, and the tendency of *Rothia mucilaginosa* increased. Comparing the thermal water before and after bathing on the species level, *Rothia mucilaginosa* significantly increased, while the tendencies for *Paracoccus aminovorans* and *Paracoccus marcusii* decreased. Comparing the tap water before and after bathing, on the genus level, the tendency for *Flavobacterium* was to increase in number. On the genus level, comparing the tap water before and after bathing, there were no significant changes. In comparing the thermal water in the control group before and after bathing, Rothia bacteria increased significantly, while there was a tendency for *Hemophilius* (*parainfuenzae*) to also increase, *Pseudomonas* had a tendency to decrease, *Neisseriae*significantly increased, and *Flavobacterium* tended to decrease. Comparing thermal water and the control group before and after bathing on the species level, *Geobacillus vulcani* decreased significantly and the tendency of *Burkholderia gladioli* was to decrease (Table 2).The main result was that the thermal water changed certain microbiomes of the skin, both on the genus and species levels, but there were no significant changes in the tap water used, either at the genus or species levels (Figure 1, Figure 2, Figure 3, Figure 4, Figure 5 and Figure 6). The first two figures represent the Shannon index, but the other two represent the Bray Curtis index, and the fifthand sixthfigures show the relative distribution of the core genera and species. No side effects during the treatment were detected.

## 4. Discussion

There were significant differences between several components of the skin microbiome of patients after they were treated with thermal water. *Deinococcus* increased significantly at the genus level after thermal bathing. *Deinococcus* was resistant to X-rays and UV rays. It is a well-known fact that *Staphylococcus aureus* is more common in patients with chronic skin inflammatory diseases. *Deinococcus* may play an important role in inhibiting *Staphylococcus aureus* infection. The proportion of *Deinocccus* correlates negatively with inflammatory skin diseases [23]. This effect is considered to have a beneficial effect. The tendency for *Rothia* was that its numbers increased. The tendency is represented by the large differences calculated on the basis of the log fold change. *Rothia dentocariosa* and *Rothia mucilaginosa* are gram-positive bacteria. These bacteria can be found in the pharynx and oral cavity [24]. After bathing in thermal water, the species level of *Rothia mucilaginosa* increased significantly. *Rothia mucilaginosa* secretes anti-inflammatory mediators, which are involved in NF-κB pathways. *Rothia mucilaginosa* is an anti-inflammatory bacterium in chronic lung diseases [25], which signifies that *Rothia mucilaginosa* is considered to have a beneficial effect. There were tendencies for *Paracoccus aminovorans* and *Paracoccus marcusii* to decrease. In healthy volunteers in the experimental group who contracted infections on the forearm, there were greater numbers of these bacteria than on normally healed skin [26]. From this, it can be deduced that there is a beneficial effect. When the temperature of the thermal water in the control group is compared before and after bathing at the genus level, *Rothia* increased significantly. *Rothia* occurs in a healthy oral cavity. There was a tendency for *Hemophilius parainfuenziae* to increase, although our study could not establish that there were pathogen strains produced, and in fact, the majority of strains could not be classified. Since *Pseudomonas* is one of the sturdiest bacteria and tends to decrease with balneotherapy treatment, a benefit can be deduced. Although there are several opportunistic bacteria, such as those in the oral cavity and genital tract, that propagate as a consequence of the conditions induced in this study, the ones found have a positive influence on the microbiome. *Neisseria* has increased significantly. There are 12 strains of *Neisseria*, but only two pathogens are known; the others are not classified, and there were no clinical manifestations in the patients. The tendency of *Flavobacterium* was that it decreased, which is considered a benefit, namely because *Flavobacterium* is also a pathogen strain. Even fewer human studies have been published on *Flavobacteria*. Their pathological role is not clear, but as gram negative bacteria, they can be the source of many infections. When the thermal water for the control group was compared before and after bathing on the species level, *Geobacillus vulcani* decreased significantly. The genus *Geobacillus* belongs to the phylum *Firmicutes. Geobacillus vulcani*, which is strongly thermophilic, can play important roles in the functions of certain enzymes [27]. Its clinical effect is unknown. The tendency was for *Burkholderia gladioli* to also decrease. There is a recently published article on skin infections caused by *Burkholderia gladioli* [28], so the decreasing effect is also considered to have beneficial effects. Several studies have been published on the composition of the microbiome of various thermal springs, some of which have analyzed the thermal springs of their respective countries [29]. 

In human clinical trials, any microbiome during balneotherapy can hardly be found. In an open study by French authors, 29 patients with psoriasis were bathed in medicinal water containing selenium, for whom the sampling was affected (plaque) and occurred from an unaffected skin area. Clinical improvement was measured by PASI. The bacterial communities examined were similar in psoriatic and non-psoriatic sites. After the thermal cure, the genus *Xanthomonas* increased significantly, and to a lesser degree, so did the genus *Corynebacterium.* This discovery is significant because it has already been documented that the *Xanthomonas* strain, which belongs to the *Xanthomonadaceae* family, which belongs to the *Proteobacteria phylum* community, is known for its keratolytic effects [30]. The diversity of microbes in psoriasis lesions is significantly greater compared to the patient’s normal skin surface than to that of a healthy control. During the Dead Sea microclimate, the bacterial and fungal nature of the skin was examined on healthy volunteers. The diversity of the bacterial community remained the same before and after the treatment, while the fungal diversity significantly decreased after the treatment [31]. The skin is an immunological organ, consisting of different mediators, e.g., keratinocytes, Langerhans cells, dendritic cells, and so on. The different elements affect the skin in various ways. When we use sulphur baths, the H_2_S is able to absorb into the skin and mucosae. The effect of sulphurous mineral waters is related mainly to sulphur’s keratolytic effects. Sulphurous mineral water has anti-inflammatory, bactericid, and antifungal properties [32]. The salty thermal water also has a strong influence on the skin’s immune system. The sodium chloride activates the induction of Th17 cells. The production of CD4^+^ T cells increases [33]. An animal model of imiquimod-induced psoriasis-like skin inflammation was demonstrated by Korean authors in Natrium chloride mineral water. They were able to prove that the mineral water balneotherapy group showed faster improvement in skin erythema than the distilled water bathing group. The mRNA levels of IL-17A and IL-23 on the lesion in the mineral water group were decreased, and IL-4 and IL-5 were also significantly decreased in the mineral water group but not in the distilled water group. Their results support the idea that balneotherapy can be used as an effective treatment for psoriasis [34]. Radon thermal water changed the immune cells (cytotoxic T and NK cells) and reduced the activation marker CD69 on T, B, and NK cells. After bathing, the HLA-DR^+^ T cells increased [35]. These studies demonstrated a close connection between balneotherapy and the immune system [36,37]. The dysbalance of the skin microbiome can lead to an altered immune system, which can lead to inflammatory diseases of the skin. In Avène Thermal Spring Water were isolated 39 *phylia* (mainly *Nitrospirae* and *Proteobacteria*) [38]. The mud can affect the microbiology of the skin, which has a beneficial effect on immune function (5 in vitro studies so far). Therefore, it would be important to investigate the interaction of different muds with the microbiome of the skin in order to better characterize their properties [39,40]. A portion of mud extracts inhibited the expression of VCAM-1 by endothelial cells and reduced monocyte adhesion to activated endothelial cells, which means an anti-inflammatory effect [41]. Water springs provide important ecosystem services, including drinking water supply, recreation, and balneotherapy, but their microbial communities remain largely unknown. In a study, authors examined the spring water microbiome of coma no terme (Italy) in the thermal spa, storage tank, and bathtubs. They found among the core microbiome *Sphingomonadales*, *Rhizobiales*, *Caulobacterales*, *Bradyrhizobiaceae*, and *Moraxellaceae*; it was concluded that aquatic microbiomes are essentially based on surface and human-associated environments [42]. Sodium hydrosulfide efficiently blocked the induction of pro-inflammatory cytokines and counterbalanced the formation of ROS in vitro. The authors found that it enhanced the release of IL-10, a potent anti-inflammatory cytokine [43].

The tendencies and significant changes that we can observe after treatment with medicinal waters but are unobservable with tap water have proven the advantageous effects of balneotherapy. Limitation: An increase in the number of patients would probably change the trends to a significant level for some microbiomes. Tests were performed with only one certain mineral water. In this study, only healthy women were included.

## 5. Conclusions

This study verifies that thermal water decreased the number of certain inflammatory infectious agents and enhanced their positive effects, which can be proven at the molecular level. 

Presumably, not only the mineral content but also the concentration has influence on the microbiome systems. We can effectively summarize that the future of balneological research is in the examination of the skin microbiome system. The research of the skin microbiome during balneotherapy can be one of the most intriguing and exciting questions of the future and can bring us closer to understanding the mechanism of action of balneotherapy.

## Figures and Tables

**Table 1:** Anorganic components of the Lakitelek thermal water(Gabriella’s Springs).

Nitrite	<0.02 mg/L
Nitrate	<1.0 mg/L
Chloride	30 mg/L
Sulfate	<5 mg/L
Ammonium	4.6 mg/L
Hydrogencarbonate	618 mg/L
Carbonate	18 mg/L
Iron	109 μg/L
Manganese	11.5 μg/L
Arsenic	6.5 μg/L
Sodium	240.3 mg/L
Potassium	3.2 mg/L
Calcium	14.1 mg/L
Magnesium	7.6 mg/L
Lithium	30.2 μg/L
Plumbum	<0.05 μg/L

**Table 2:** Changes in core species and genus abundances detected by DESeq2. A difference between treatments was considered significant if the Benjamini-Hochberg corrected *p*-value was below 0.05 (column Significant). We have regarded a difference to show a change in tendency without being significant when only the uncorrected *p*-value was below 0.05 (column Tendency).

	Significant Level	Tendency
Core genus diff. abundance after thermal vs. Control	Rothia increased Neisseriae increased	Hemophilius parainfuenzia increased Pseudomonas decreased Flavobacterium decreased
Core genus diff. abundance thermal after vs. before	Deinococcus increased	Rothia mucilaginosa increased
Core species diff.abundance thermal after vs. before	Rothia mucilaginosa increased	Paracoccus aminovorans and Paracoccus marcusii decreased
Cores species diff. abundance after thermal vs. control	Geobacillus vulcanii decreased	Burkholderia gladioli decreased
Core genus diff. abundance Control after vs. before	No change	Flavobacterium increased
Core species diff. abundance control after vs. before	No change	No change

## Data Availability

Data are available upon reasonable request to the corresponding author.

## Abbreviations

Nex Generation Sequencing (NGS); Psoriasis Activity Index (PASI); Ultraviolet (UV); Deoxyribonucleic Acid(DNA); Non-metric Multidimensional Scaling (NMDS); Messenger RNA(mRNA); HumanLeukocyteAntigen—DRisotype(HLA-DR); T helper*17*cells (Th-*17*); Interleukin 17 (IL-17); Hydrogen sulfide(H2S); *Reactive oxygen species*(*ROS*); Deoxyribonucleic acid(DNA).
